# Higher resonance schemes and Koszul modules of simplicial complexes

**DOI:** 10.1007/s10801-024-01313-2

**Published:** 2024-03-29

**Authors:** Marian Aprodu, Gavril Farkas, Claudiu Raicu, Alessio Sammartano, Alexander I. Suciu

**Affiliations:** 1 Simion Stoilow Institute of Mathematics, P.O. Box 1-764, 014700 Bucharest, Romania; 2 Faculty of Mathematics and Computer Science, University of Bucharest, Bucharest, Romania; 3 Institut für Mathematik, Humboldt-Universität zu Berlin, Unter den Linden 6, 10099 Berlin, Germany; 4 Department of Mathematics, University of Notre Dame, 255 Hurley, Notre Dame, IN 46556 USA; 5 Dipartimento di Matematica, Politecnico di Milano, Via Bonardi 9, 20133 Milan, Italy; 6 Department of Mathematics, Northeastern University, Boston, MA 02115 USA

**Keywords:** Simplicial complex, Square-free monomial ideal, Koszul module, Resonance variety, Reduced scheme, Hilbert series, Primary 13F55, Secondary 14M12, 16E05

## Abstract

Each connected graded, graded-commutative algebra *A* of finite type over a field $\Bbbk$ of characteristic zero defines a complex of finitely generated, graded modules over a symmetric algebra, whose homology graded modules are called the *(higher) Koszul modules* of *A*. In this note, we investigate the geometry of the support loci of these modules, called the *resonance schemes* of the algebra. When $A=\Bbbk \langle \Delta \rangle$ is the exterior Stanley–Reisner algebra associated to a finite simplicial complex $\Delta$, we show that the resonance schemes are reduced. We also compute the Hilbert series of the Koszul modules and give bounds on the regularity and projective dimension of these graded modules. This leads to a relationship between resonance and Hilbert series that generalizes a known formula for the Chen ranks of a right-angled Artin group.

## Introduction and statement of results

Koszul modules are graded modules over a symmetric algebra that are constructed from the classical Koszul complex. They emerged from geometric group theory and topology [2, 12] and found applications in other fields such as algebraic geometry. One prominent instance is [1], where the effective vanishing in high degrees of some Koszul modules led to a new proof of the celebrated Green’s Conjecture on syzygies of generic canonical curves. The argument relies on a connection between the graded pieces of those particular Koszul modules and the Koszul cohomology of the tangent developable surface of a rational normal curve. The non-trivial vehicle that permits the passage in [1] from symmetric powers (Koszul modules) to exterior powers (Koszul cohomology) is an explicit version of the Hermite reciprocity formula.

It is the aim of this paper to describe a completely new instance where the passage from Koszul modules to Koszul cohomology of some homogeneous coordinate ring is still possible. The setup is, however, simpler and more elementary than the one involved with Green’s Conjecture.

For a ground field $\Bbbk$ of characteristic 0, a classical construction of Stanley and Reisner associates to every simplicial complex $\Delta$ on *n* vertices a graded, graded-commutative algebra $\Bbbk \langle \Delta \rangle = E/J_{\Delta }$, where $E={\bigwedge }_{\Bbbk }(e_1,\dots , e_n)$ is the exterior algebra over $\Bbbk$ and $J_{\Delta }$ is the ideal generated by all the monomials $e_{\sigma }=e_{j_1}\wedge \cdots \wedge e_{j_s}$ corresponding to simplices $\sigma =(j_1,\dots , j_s)$ with $1\leqslant j_1<\cdots <j_s \leqslant n$ which do not belong to $\Delta$.

Let $S:=\Bbbk [x_1,\ldots , x_n]$ be the polynomial ring in *n* variables over $\Bbbk$, and consider the cochain complex $(\Bbbk \langle \Delta \rangle ^{\bullet }\otimes _{\Bbbk } S, \delta )$ of free, finitely generated, graded *S*-modules obtained by applying the BGG correspondence to the finitely generated, graded *E*-module $\Bbbk \langle \Delta \rangle ^{\bullet }$. The Fitting ideals of this complex define the *jump resonance loci* of our simplicial complex,

1.1 $$\begin{aligned} \mathcal {R}^i(\Delta ):=V\left( {\text {Fitt}}_{\beta _{i+1}} (\delta ^{i-1}\oplus \delta ^{i})\right) , \end{aligned}$$

where $\beta _{i+1}$ is the number of faces of dimension *i* in $\Delta$. It was shown in [10] that the irreducible components of $\mathcal {R}^i(\Delta )$ are coordinate subspaces of $\Bbbk \langle \Delta \rangle ^1=\Bbbk ^n$, given explicitly in terms of the (simplicial) homology groups of certain subcomplexes of $\Delta$.

Now let $\bigl (\Bbbk \langle \Delta \rangle _{\bullet } \otimes _{\Bbbk } S, \partial \bigr )$ be the dual chain complex, and define the *Koszul modules* (in weight *i*) of the simplicial complex $\Delta$ to be the homology *S*-modules of this complex,

1.2 $$\begin{aligned} W_i(\Delta ):=H_i\bigl (\Bbbk \langle \Delta \rangle _{\bullet } \otimes _{\Bbbk } S, \partial \bigr ). \end{aligned}$$

An alternate definition of resonance is given by the support loci of these modules,

1.3 $$\begin{aligned} \mathcal {R}_i(\Delta ) :=V\bigl ({\text {Ann}}(W_i(\Delta ))\bigr ). \end{aligned}$$

These varieties, called the *support resonance loci*, are again finite unions of coordinate subspaces. Though they do not coincide in general with the previously defined sets $\mathcal {R}^i(\Delta )$, it is known that $\mathcal {R}_1(\Delta )=\mathcal {R}^1(\Delta )$ (away from 0) and $\bigcup _{j\leqslant i}\mathcal {R}_j(\Delta )=\bigcup _{j\leqslant i} \mathcal {R}^j(\Delta )$ for all $i\geqslant 1$.

A notable property of the higher Koszul modules associated to simplicial complexes is that they are multigraded as opposed to the general case when they are only graded modules. Using the general theory of multi-graded square-free modules, we prove that the multi-graded pieces of the Koszul modules can be described as multi-graded pieces of some ${\text {Tor}}$’s over symmetric algebras. It is known (see, for example, [12]) that the graded pieces of weight-one Koszul modules are graded pieces of ${\text {Tor}}$’s over exterior algebras; however, their relations with ${\text {Tor}}$’s over symmetric algebras are quite rare in general.

### Theorem 1.1

For any $i\geqslant 1$ and any square-free multi-index $\textbf{b}$, there is a natural isomorphism of vector spaces,

1.4 $$\begin{aligned} \left[ W_i(\Delta )\right] _\textbf{b} \cong \left[ {\text {Tor}}^S_{|\textbf{b}|-i} (\Bbbk , \Bbbk [\Delta ])\right] _\textbf{b}^\vee , \end{aligned}$$

where $\Bbbk [\Delta ]$ is the polynomial Stanley–Reisner ring of $\Delta$.

We refer to Sect. 3.1 for a quick review of multi-graded square-free modules. This multigraded structure of the Koszul modules is captured in the Hilbert series.

### Theorem 1.2

For every simplicial complex $\Delta$, the multigraded Hilbert series of the Koszul modules $W_i(\Delta )$ are given by

$$\begin{aligned} \sum _{\textbf{a}\in \mathbb {N}^n} \dim _{\Bbbk } [W_i(\Delta )]_\textbf{a} \, \textbf{t}^\textbf{a}= \sum _{\begin{array}{c} \textbf{b}\,\in \, \mathbb {N}^n\\ \textbf{b} {\text {square-free}} \end{array}} \dim _{\Bbbk }({\widetilde{H}}_{i-1}(\Delta _\textbf{b}; \Bbbk )) \frac{\textbf{t}^\textbf{b}}{\prod _{j \in {\text {Supp}}(\textbf{b})}(1-t_j)}. \end{aligned}$$

In Sect. 5, we give a precise description of the irreducible components of the support resonance loci. In each weight *i*, they correspond to maximal subcomplexes with non-vanishing reduced homology in degree $i-1$.

### Theorem 1.3

For every simplicial complex $\Delta$ and every $i\geqslant 1$, the scheme structure on the support resonance $\mathcal {R}_i(\Delta )$ is reduced. Moreover, the decomposition in irreducible components is given by

1.5 $$\begin{aligned} \mathcal {R}_i(\Delta )=\bigcup _{\begin{array}{c} \mathsf {V'}\subseteq {\textsf{V}}\, \mathrm { maximal\, with }\\ {\widetilde{H}}_{i-1}(\Delta _{\mathsf {V'}};\Bbbk )\ne 0 \end{array}}\Bbbk ^{\mathsf {V'}}. \end{aligned}$$

Particularly interesting is the case when $\Delta$ is 1-dimensional, that is, it may be viewed as a finite simple graph $\Gamma$. It was shown in [9] that all the irreducible components of $\mathcal {R}^1(\Gamma )$ are coordinate subspaces, which correspond to the maximally disconnected full subgraphs of $\Gamma$. This result comes as a direct consequence of our analysis. The statement concerning the reducedness of $\mathcal {R}_i(\Delta )$ can be compared with the detailed study performed in [3] on the scheme structure of (support) resonance varieties associated to classical Koszul modules.

## Graded algebras, Koszul modules, and higher resonance

We start in a more general context (adapted from the setup in [11, 13]) that will be used throughout the paper.

### Chain complexes associated to graded algebras

Let $A^{\bullet }$ be a graded, graded-commutative algebra over a field $\Bbbk$ of characteristic 0, with multiplication maps $A^i \otimes _{\Bbbk } A^j \rightarrow A^{i+j}$. We will assume that *A* is connected (that is, $A^0=\Bbbk$) and of finite type (that is, $\dim _{\Bbbk }A^i<\infty$, for all $i>0$), and we will write $\beta _i(A)=\dim _{\Bbbk }A^i$. To avoid trivialities, we always assume that $\beta _1(A)\ne 0$.

For each $a\in A^1$, graded commutativity of multiplication yields $a^2=0$; therefore, we have a cochain complex 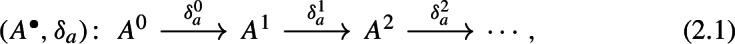 with differentials $\delta ^i_{a} (u)= a \cdot u$, for all $u \in A^i$. The *resonance varieties* of *A* are the jump loci for the cohomology groups of this complex: for each $i\geqslant 0$, we put

2.2 $$\begin{aligned} \mathcal {R}^i(A):=\bigl \{a \in A^1 \mid H^i(A^{\bullet }, \delta _{a}) \ne 0 \bigr \}. \end{aligned}$$

Clearly, these are homogeneous subsets of the affine space $A^1$. Since $A^0$ is 1-dimensional, generated by $1\in \Bbbk$, and since $\delta _a(1)=a$ for each $a\in A^1$, it follows that $\mathcal {R}^0(A)=\{0\}$. The most studied is the first resonance variety, which can be described as the set

2.3 $$\begin{aligned} \mathcal {R}^1(A) =\{ a \in A^1 \mid \exists b \in A^1, \; 0\ne a\wedge b \in K^{\perp } \} \cup \{0\}, \end{aligned}$$

where $K^{\perp }$ denotes the kernel of the multiplication map $A^1\wedge A^1 \rightarrow A^2$.

Let us now fix a $\Bbbk$-basis $\{ e_1,\dots , e_n \}$ of $A^1$, and let $\{ x_1,\ldots , x_n \}$ be the dual basis of the dual $\Bbbk$-vector space $A_1=(A^1)^{\vee }$. This allows us to identify the symmetric algebra ${\text {Sym}}(A_1)$ with the polynomial ring $S=\Bbbk [x_1,\dots , x_n]$, the coordinate ring of the affine space $A^1\cong \Bbbk ^{\beta _1(A)}$.

Viewing $A^{\bullet }$ as a graded module over the exterior algebra $E^{\bullet }={\bigwedge }A^1$, the BGG correspondence [7] yields a cochain complex of finitely generated, free *S*-modules, 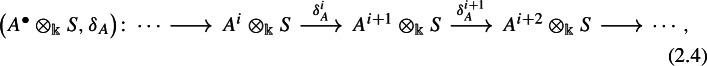 whose coboundary maps are the *S*-linear maps given by $\delta ^{i}_A(u \otimes s)= \sum _{j=1}^{n} e_j u \otimes s x_j$ for $u\in A^i$ and $s\in S$. It is readily seen that this cochain complex is independent of the choice of basis for $A^1$ and that, moreover, the specialization of $(A\otimes _{\Bbbk } S,\delta _A)$ at an element $a\in A^1$ coincides with the complex $(A,\delta _a)$ defined by (2.1).

#### Remark 2.1

Typically, the elements in the exterior algebra $E^{\bullet }$ are given negative degrees, see, for instance, [7]. However, we prefer to work here with the positive grading, which amounts to negating the standard action of the torus $(\Bbbk ^*)^n$ on $E^{\bullet }$ as well. This convention carries over to the exterior Stanley–Reisner rings $A^{\bullet }=\Bbbk \langle \Delta \rangle$ in Sect. 3.2 and is compatible with the notation of [4], as recalled in Proposition 3.8.

It follows directly from the definition (2.2) that a point $a \in A^1$ belongs to $\mathcal {R}^i(A)$ if and only if ${\text {rank}}\delta ^{i-1}_a + {\text {rank}}\delta ^{i}_a < \beta _i(A)$. Therefore,

2.5 $$\begin{aligned} \mathcal {R}^i(A)= V \big ( {\text {Fitt}}_{\beta _{i+1}(A)} \big (\delta ^{i-1}_A\oplus \delta ^{i}_A\big ) \big ), \end{aligned}$$

where $\psi _1\oplus \psi _2$ denotes the block sum of two matrices, ${\text {Fitt}}_r(\psi )$ denotes the ideal of minors of size $n-r$ of a matrix $\psi :S^m\rightarrow S^n$, and *V*(*I*) denotes the zero-set of an ideal $I\subset S$. This shows that the sets $\mathcal {R}^i(A)$ are algebraic subvarieties of the affine space $A^1$ called *jump resonance loci*.

### Koszul modules and their support loci

Set $A_i:=(A^i)^{\vee }$ and $\partial ^A_i:=(\delta _A^{i-1})^{\vee }$ and consider the chain complex of finitely generated *S*-modules 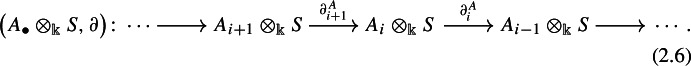 We define the *Koszul modules (in weight i)* of the algebra *A* as the homology *S*-modules of this chain complex, that is,

2.7 $$\begin{aligned} W_i(A) :=H_i\bigl (A_{\bullet }\otimes _{\Bbbk } S\bigr ) . \end{aligned}$$

Clearly, these are finitely generated, graded *S*-modules. The degree *d* component of the Koszul module $W_i(A)$ is computed by the homology of the complex 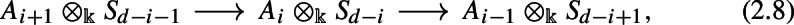 where we recall that $S={\text {Sym}}(A^1)$. It follows straight from the definitions that $W_0(A)=\Bbbk$ is the trivial *S*-module.

Setting $E_{\bullet }:={\bigwedge }A_1$, the first Koszul module also has the following presentation 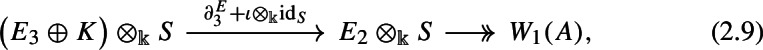 where 

The *resonance schemes* of the graded algebra *A* are defined by the annihilator ideals of the Koszul modules of *A*,

2.10 $$\begin{aligned} \mathcal {R}_i(A) :={\text {Spec}}\bigl (S/ {\text {Ann}}W_i(A)\bigr ). \end{aligned}$$

By slightly abusing notation, we also denote by $\mathcal {R}_i(A)={\text {Supp}}W_i(A)$ the underlying sets and call them *support resonance loci*. Note that the algebra structure on $A^{\bullet }$ is not essential in the discussion above, as the definitions of Koszul modules and support resonance loci only use the *E*-module structure. In particular, the constructions apply for finitely generated graded *E*-modules, as well.

Clearly, $\mathcal {R}_0(A)=\mathcal {R}^0(A)=\{0\}$. More generally, suppose $W_j(A)\ne 0$ for all $1\leqslant j\leqslant i$. Then, as shown in [11, Theorem 2.5], the support resonance loci are related to the jump resonance loci by the formula^1^

2.11 $$\begin{aligned} \bigcup _{j\leqslant i} \mathcal {R}_j(A) = \bigcup _{j\leqslant i} \mathcal {R}^j(A). \end{aligned}$$

In particular, if $W_1(A)\ne 0$, then $\mathcal {R}_1(A) =\mathcal {R}^1(A)$.

### Quotients of exterior algebras through ideals generated in fixed degree

We now discuss a particularly interesting case of this general construction. Fix integers $d\geqslant 1$ and $n\geqslant 3$. Let *V* be an *n*-dimensional vector space over the field $\Bbbk$, and let $K\subseteq {\bigwedge }^{d+1}V$ be a subspace. Set $S:={\text {Sym}}(V)$ and $E^\bullet :={\bigwedge }V^\vee$, and then consider the linear subspace

2.12 $$\begin{aligned} K^\perp :=\big ( {\bigwedge }^{d+1}V/K \big )^\vee = \big \{\varphi \in {\bigwedge }^{d+1}V^\vee \mid \varphi _{|K}= 0\big \} \subseteq {\bigwedge }^{d+1}V^\vee . \end{aligned}$$

Letting $A^\bullet :=E^\bullet /\langle K^\perp \rangle$ be the quotient of the exterior algebra $E^\bullet$ by the (homogeneous) ideal generated by $K^\perp$, we clearly have $K=A_{d+1}$. Conversely, if $J\subseteq E^\bullet$ is a homogeneous ideal generated in degree $d+1$ and we take $K^\perp :=J_{d+1}$, then the algebra $A^\bullet =E^\bullet /J$ is obtained as above. Denote by *j* the inclusion of the dual algebra $A_\bullet$ into $E_\bullet$. Recalling that $\partial _i :{\bigwedge }^i V\otimes _{\Bbbk } S\rightarrow {\bigwedge }^{i-1} V\otimes _{\Bbbk } S$ is the Koszul differential, we have the following characterization.

#### Proposition 2.2

The Koszul modules $W_i(V,K)=W_i(A)$ satisfy the following properties:

1. $$W_i(A)=0$$ for $$i\leqslant d-1$$.
2. $$W_d(A)={\text {coker}}\big (\partial _{d+2}+j_{d+1} \otimes _{\Bbbk } S\big )$$.

#### Proof

The first part is quite straightforward, as $J_{i=0}$ for $i\leqslant d-1$ and hence $A_i=E_i$ for $i\leqslant d-1$. For the second part, first note that the *d*-th Koszul module is in this case the middle homology of the complex 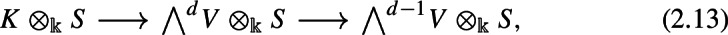 and hence 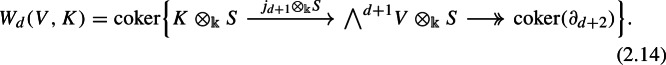 Applying now the Snake Lemma to the diagram 
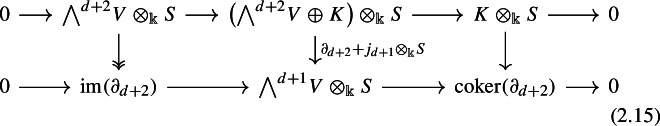
 establishes the claim. $\square$

#### Remark 2.3

Note that the Snake Lemma also applies to the diagram 
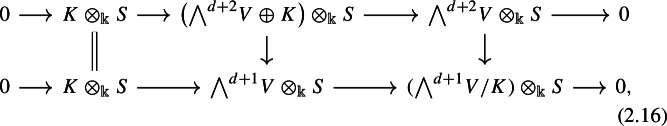
 leading to the simpler presentation 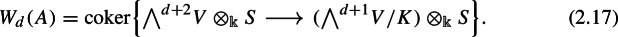


If $d=1$, in weight 1 we recover the original Koszul module *W*(*V*, *K*) of a pair (*V*, *K*) with $K\subseteq {\bigwedge }^2V$ considered in [2, 3, 12] and elsewhere. However, note the shift by two in degrees, that is, $W(V,K)=W_1(A)(2)$.

#### Example 2.4

Let *X* be a smooth complex projective variety, and consider a vector bundle *E* on *X* of rank $\geqslant r+1$, for some integer $r\geqslant 1$. We consider the determinant maps 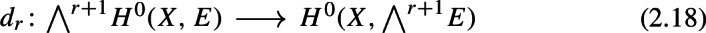 and take $K_r^\perp :=\ker (d_r)$. Then the above construction applies, producing for each *r* a series of Koszul modules $W_r(X,E):=W_r\bigl (H^0(X, E)^{\vee }, K_r \bigr )$.

As in the case $d=1$ (see [1, 12]), we have a geometric characterization of vanishing resonance, in which case the corresponding Koszul module is of finite length and hence vanishes in high degrees. For an element $\omega \in {\bigwedge }^{d+1}V^\vee$, we denote by $\varphi (\omega ):V^\vee \rightarrow {\bigwedge }^{d+2}V^\vee$ the map $a\mapsto a\wedge \omega$. Consider the projective variety parameterizing the decomposable elements,

2.19 $$\begin{aligned} \Sigma _d:=\left\{ [\omega ]\in \mathbb {P} \big ({\bigwedge }^{d+1}V^\vee \big ) \mid {\text {rank}}(\varphi (\omega ))\leqslant n-1\right\} . \end{aligned}$$

Standard multilinear algebra proves the following proposition.

#### Proposition 2.5

If $d\geqslant 2$, then $\mathcal {R}_d(A)=\{0\}$ if and only if $\mathbb {P}(K^\perp )\cap \Sigma _d=\emptyset$.

#### Proof

Recall that $a\in V^\vee$ divides $\omega$ if and only if $a\wedge \omega =0$. Therefore, $\mathcal {R}_d(A)\ne \{0\}$ if and only if there exist $a\in V^\vee$ and $b\in {\bigwedge }^dV^\vee$ such that $0\ne a\wedge b\in K^\perp$. This is equivalent to the existence of a nonzero element $a\in V^\vee$ and of a nonzero element $\omega \in K^\perp$ such that $a\wedge \omega =0$, i.e., $0\ne a\in \ker (\varphi (\omega ))$, and hence $[\omega ]\in \Sigma _d$. $\square$

The case $d=1$ is special, since $\varphi (\omega )$ non-injective implies its kernel is at least 2-dimensional. Indeed, if $\omega =a\wedge b\ne 0$ then $\ker (\varphi (\omega ))$ is generated by *a* and *b*. In this case, $\Sigma _1$ is the Grassmann variety ${\text {Gr}}_2(V^\vee )\subseteq \mathbb {P}\bigl ({\bigwedge }^2 V^{\vee }\bigr )$.

#### Remark 2.6

For the Koszul module $W_r(X,E)$ considered in Example 2.4, we have that the resonance $\mathcal {R}_r(X,E):={\text {Supp}}W_r(X,E)$ is non-trivial if and only if there exists a section $0\ne s\in H^0(X,E)$ such that the determinant map $d_s:{\bigwedge }^r H^0(X,E)\rightarrow {\bigwedge }^{r+1} H^0(X,E)$ given by $\omega \mapsto d_{r+1}(s\wedge \omega )$ is not injective.

## Simplicial complexes and their Koszul modules

### Square-free modules

We start this section with some algebraic preliminaries regarding square-free modules. We recall from [14] some basic facts about this type of modules, which will be needed in Sects. 4.2, 5, and 6.1.

Let *V* be a $\Bbbk$-vector space of dimension *n*, and identify the symmetric algebra ${\text {Sym}}(V)$ with the polynomial ring $S=\Bbbk [x_1,\ldots ,x_n]$. We consider the standard $\mathbb {N}^n$-multigrading on *S*, defined by $\deg (x_i) = \textbf{e}_i \in \mathbb {N}^n$, where $\textbf{e}_i=(0,\ldots ,1,\ldots ,0)$ is the multi-index with 1 placed in the *i*-th position. Given a multi-index $\textbf{a}=(a_1, \ldots , a_n) \in \mathbb {N}$, its support is defined as the set ${\text {Supp}}(\textbf{a}):=\{ i \mid a_i > 0\}$.

#### Definition 3.1

An $\mathbb {N}^n$-graded *S*-module *M* is said to be *square-free* if for any $\textbf{a}\in \mathbb {N}^n$ and any $i\in {\text {Supp}}(\textbf{a})$, the multiplication map 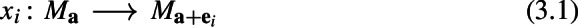 is an isomorphism.

This definition is a direct generalization of the case of ideals. Indeed, an ideal $I \subseteq S$ is a square-free module if and only if it is a square-free monomial ideal, and this is also equivalent to *S*/*I* being a square-free module.

Note that a free $\mathbb {N}^n$-graded *S*-module is square-free if and only it is generated in square-free multidegrees.

#### Proposition 3.2

If $f:M\rightarrow N$ is a morphism of $\mathbb {N}^n$-graded *S*-modules, and *M* and *N* are square-free modules, then $\ker (f)$ and ${\text {coker}}(f)$ are also square-free. Moreover, if 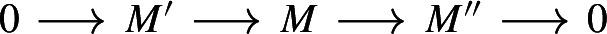 is an exact sequence of $\mathbb {N}^n$-graded *S*-modules, and $M'$ and $M''$ are square-free, then so is *M*.

Proposition 3.2 has a few interesting consequences.

#### Corollary 3.3

Let *M* be an $\mathbb {N}^n$-graded square-free *S*-module. Then all the modules in the minimal free $\mathbb {N}^n$-graded resolution of *M* are square-free.

#### Corollary 3.4

If $\textbf{F}$ is a bounded complex of free square-free *S*-modules, then the homology modules of $\textbf{F}$ are also square-free.

The following result will be of particular interest for us.

#### Theorem 3.5

If *M* is an $\mathbb {N}^n$-graded, square-free *S*-module, then its annihilator is a square-free monomial ideal. In particular, the annihilator of *M* is a radical ideal.

#### Proof

Since *M* is an $\mathbb {N}^n$-graded *S*-module, the annihilator ${\text {Ann}}(M) \subseteq S$ is also $\mathbb {N}^n$-graded, that is, it is a monomial ideal. Let $m=x_{1}^{a_1}\cdots x_{n}^{a_n}\in {\text {Ann}}(M)$ be a monomial annihilating *M*, and assume $a_k>1$ for some *k*. Then the multiplication map 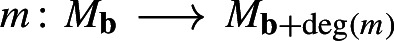 is zero for all $\textbf{b} \in \mathbb {N}^n$. We have $k \in {\text {Supp}}(\textbf{b}+\deg (m)-\textbf{e}_k)$, and so, by hypothesis, the map 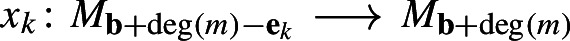 is an isomorphism. Therefore, 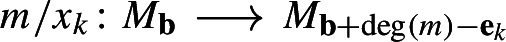 is the zero map for all $\textbf{b} \in \mathbb {N}^n$, and thus, $x_{1}^{a_1}\cdots x_k^{a_k-1}\cdots x_{n}^{a_n}\in {\text {Ann}}(M)$. By repeating the argument, we see that ${\text {Ann}}(M)$ is a square-free monomial ideal. $\square$

Finally, we note that Theorem 3.5 and [14, Lemma 2.2] give the following.

#### Proposition 3.6

Let *M* be a finitely generated $\mathbb {N}^n$-graded, square-free *S*-module. Then the annihilator scheme structure on the support of *M* is reduced. Moreover, the decomposition of the support in irreducible components is given by

$$\begin{aligned} {\text {Supp}}(M)=\bigcup _{\begin{array}{c} \textbf{b}\,\text {square-free}\\ \text {maximal with}\, M_\textbf{b}\ne 0 \end{array} }\Bbbk ^{{\text {Supp}}(\textbf{b})}, \end{aligned}$$

where $\Bbbk ^\mathsf {V'}$ denotes the locus $V(x_i|\ i\not \in {\textsf{V}}')$.

Proposition 3.6 will be essential for describing the components of the support resonance loci of a simplicial complex in the next section.

We end this section with the following definition:

#### Definition 3.7

For an $\mathbb {N}^n$-graded vector space *M*, the *square-free part* of *M* is the subspace ${\text {sqf}}(M)\subseteq M$ concentrated in square-free multidegrees.

### Stanley–Reisner rings

Let $S=\Bbbk [x_1,\dots , x_n]$ be the polynomial ring in *n* variables over a field $\Bbbk$ of characteristic 0. Given a simplicial complex $\Delta$ on *n* vertices, we let $\Bbbk [\Delta ] :=S/I_{\Delta }$ be the (polynomial) *Stanley–Reisner ring* of $\Delta$, where $I_{\Delta }$ is the ideal generated by the (square-free) monomials $x_{\sigma }=x_{i_1} \cdots x_{i_s}$ for all simplices $\sigma =(i_1,\dots , i_s)$ with $1\leqslant i_1<\cdots <i_s \leqslant n$ not in $\Delta$. Similarly, we define the *exterior Stanley–Reisner ring* of $\Delta$ as $\Bbbk \langle \Delta \rangle :=E/J_{\Delta }$, where $E={\bigwedge }(e_1,\dots , e_n)$ is the exterior algebra in *n* variables over $\Bbbk$ and $J_{\Delta }$ is the ideal generated by the monomials $e_{\sigma }=e_{i_1}\wedge \cdots \wedge e_{i_s}$ for all simplices $\sigma \notin \Delta$.

Consider the graded, graded-commutative $\Bbbk$-algebra $A^{\bullet }:=\Bbbk \langle \Delta \rangle$. As mentioned in Remark 2.1, this algebra is given the positive grading. In each degree *d*, the vector space $A^d$ is spanned by multivectors $e_\sigma$, where $\sigma$ is a $(d-1)$-dimensional face of $\Delta$. Indeed, since $\sigma =(i_1,\ldots ,i_s)\not \in \Delta$ implies $(i_1,\ldots ,i_s,j)\not \in \Delta$ for all $j\not \in {\text {Supp}}(\Delta )$, it follows that in each degree *d*, the vector space $J_{\Delta ,d}$ is spanned by the multivectors $e_\sigma$ with $\sigma \not \in \Delta$ of dimension $d-1$. With the notation of the previous sections, the dual $A_d$ is generated by the vectors $v_\sigma$ with $\sigma \in \Delta$ being of dimension $d-1$.

For an element $a=\sum _{i=1}^{n} \lambda _i e_i\in A^1$, let $(A^{\bullet }, \delta _a)$ be the cochain complex from (2.1). As shown in [4, Proposition 4.3] (see also [10, Lemma 3.4]), this complex depends only on ${\text {Supp}}(a):=\{i\mid \lambda _i\ne 0\}$; more precisely, $(A^{\bullet }, \delta _a)$ is isomorphic to $(A^{\bullet }, \delta _{\bar{a}})$, where $\bar{a}=\sum _{i\in {\text {Supp}}(a)} e_i$. The following Hochster-type formula from [4, Proposition 4.3], suitably interpreted and corrected in [10, Proposition 3.6], describes the cohomology groups of the cochain complexes $(A^{\bullet }, \delta _a)$.

#### Proposition 3.8

[4, 10] Let $\Delta$ be a finite simplicial complex on vertex set ${\textsf{V}}=[n]$ and $a\in A^1$ as above. Writing ${\textsf{V}}'={\text {Supp}}(a)$, we have

$$\begin{aligned} \dim _{\Bbbk } H^{i}\bigl (\Bbbk \langle \Delta \rangle ,\delta _a\bigr )=\sum _{\sigma \in \Delta _{{\textsf{V}}\setminus {\textsf{V}}'}} \dim _{\Bbbk } {\widetilde{H}}_{i-1-|\sigma |} \bigl ({\text {lk}}_{\Delta _{{\textsf{V}}'}}(\sigma ); \Bbbk \bigr ). \end{aligned}$$

Here $\Delta _{{\textsf{V}}'}:=\{\tau \in \Delta \mid \tau \subset {\textsf{V}}'\}$ is the simplicial complex obtained by restricting $\Delta$ to ${\textsf{V}}'$ and ${\text {lk}}_{\Delta _{{\textsf{V}}'}}(\sigma ):=\{\tau \in \Delta _{{\textsf{V}}'} \mid \tau \cup \sigma \in \Delta \}$ is the link of a simplex $\sigma$ in $\Delta _{V'}$. The range of summation in the above formula includes the empty simplex, with the convention that $|\emptyset |=0$ and ${\widetilde{H}}_{-1}(\emptyset ; \Bbbk )=\Bbbk$.

### Koszul modules of a simplicial complex

Fix a basis $v_1, \ldots , v_n$ of the $\Bbbk$-vector space *V*. Let $\textbf{K}_\bullet$ denote the Koszul complex of $x_1, \ldots , x_n$, whose *i*-th free module is $\textbf{K}_i = {\bigwedge }^i V \otimes _{\Bbbk } S$, and set $\deg (v_i) = \textbf{e}_i \in \mathbb {N}^n$. Then $\textbf{K}_\bullet$ is a complex of $\mathbb {N}^n$-graded square-free *S*-modules.

A simplicial complex $\Delta$ on vertex set $[n]=\{1, \ldots , n\}$ determines a subcomplex $\textbf{K}^\Delta _\bullet$ of $\textbf{K}_\bullet$, whose *i*-th module $\textbf{K}_i^\Delta$ is the free *S*-module generated by the exterior monomials $v_{j_1}\wedge \cdots \wedge v_{j_i}$ such that $\{j_1, \ldots , j_i\}$ is a face of $\Delta$. Applying (2.7), the *i*-th *Koszul module* $W_i(\Delta )$ defined as the *i*-th Koszul module of the exterior Stanley–Reisner ring of $\Delta$ is the *i*-th homology $H_i(\textbf{K}^\Delta _\bullet )$.

#### Proposition 3.9

For every simplicial complex $\Delta$ on *n* vertices and for every *i*, the Koszul module $W_i(\Delta )$ is an $\mathbb {N}^n$-graded square-free *S*-module.

#### Proof

The subcomplex $\textbf{K}^\Delta _\bullet$ is a complex of $\mathbb {N}^n$-graded square-free *S*-modules. By Corollary 3.4, it follows that each homology vector space $W_i(\Delta )$ is a square-free *S*-module. $\square$

## Hilbert series for Koszul modules of simplicial complexes

We fix some notation first. For a multidegree $\textbf{b}$, we denote the sum of its entries by $|\textbf{b}|$. For a square-free multidegree $\textbf{b}\in \mathbb {N}^n$, we denote by $\Delta _\textbf{b}$ the restriction of the simplicial complex $\Delta$ to the subset of the vertices ${\text {Supp}}(\textbf{b}) \subseteq \{1, \ldots , n \}$.

We denote by $\tilde{h}_i(-; \Bbbk )$ and $\tilde{h}^i(-; \Bbbk )$ the dimensions of the simplicial homology groups $\tilde{H}_i(-; \Bbbk )$ and of the reduced cohomology groups $\tilde{H}^i(-; \Bbbk )\cong \tilde{H}_i(-; \Bbbk )^\vee$ with coefficients in $\Bbbk$, respectively.

### Koszul modules versus Koszul (co)homology

We establish a duality result between the Koszul modules associated to a simplicial complex and Koszul (co)homology of the symmetric Stanley–Reisner algebra.

#### Theorem 4.1

For any $i\geqslant 1$ and any square-free multi-index $\textbf{b}$, there are natural isomorphisms of vector spaces

4.1 $$\begin{aligned} \left[ W_i(\Delta )\right] _\textbf{b} \cong \left[ {\text {Tor}}^S_{|\textbf{b}|-i} (\Bbbk , \Bbbk [\Delta ])\right] _\textbf{b}^\vee \cong {\widetilde{H}}^{i-1}(\Delta _\textbf{b}; \Bbbk )^\vee \cong {\widetilde{H}}_{i-1}(\Delta _\textbf{b}; \Bbbk ). \end{aligned}$$

#### Proof

For the square-free multidegree $\textbf{b}$, we denote $j:=|\textbf{b}| - i$.

We start by proving the first isomorphism. We use the notation from Sect. 3.2. Let $A_d\subseteq {\bigwedge }^d V$ be the subspace generated by the exterior monomials $v_\sigma$ such that $\sigma$ is a face of $\Delta$. Denote by $S_d$ the graded component of $S={\text {Sym}}(V)$ of total degree *d*. Then, the vector space $[W_i(\Delta )]_\textbf{b}$ is the middle homology of the complex of vector spaces 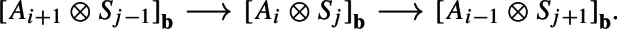 By Proposition 3.9, this complex is the same as 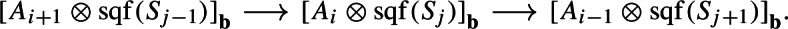 Upon identifying ${\text {sqf}}(S_{d})= \bigwedge ^d V^\vee$ via the $\Bbbk$-linear map given by $x_{i_1}\cdots x_{i_d}\mapsto e_{i_1}\wedge \cdots \wedge e_{i_d}$, this chain complex may be written as 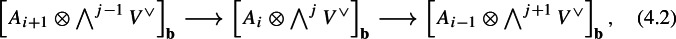 which, by dualization gives 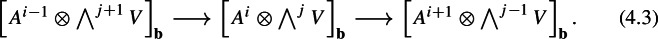 As usual, all these identifications are made without changing the positivity of the grading. After having identified $A^d = {\text {sqf}}(S/I_\Delta )_d$, the sequence (4.3) may be written as 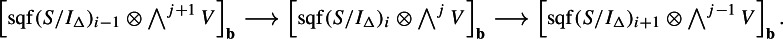
Since $\textbf{b}$ is a square-free multidegree, this complex is the same as 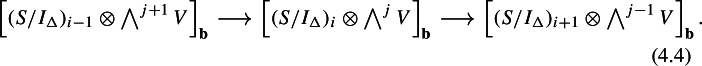
By the properties of the Koszul complex, the middle cohomology of this complex is isomorphic to

$$\begin{aligned} \left[ {\text {Tor}}^S_{j} (\Bbbk , \Bbbk [\Delta ])\right] _\textbf{b}, \end{aligned}$$

and this concludes the proof of the first isomorphism.

For the second isomorphism, note that $\left[ {\text {Tor}}^S_j(\Bbbk , \Bbbk [\Delta ])\right] _\textbf{b}$ is isomorphic to $\left[ {\text {Tor}}^S_{j-1}(\Bbbk , I_\Delta )\right] _\textbf{b}$. Indeed, for $j\geqslant 2$, this is clear, whereas for $j=1$ this follows from the fact the $I_\Delta$ is contained in the ideal generated by the variables, and hence $\Bbbk \cong \Bbbk \otimes _S \Bbbk [\Delta ]$. From [8, Proof of Theorem 8.1.1] we obtain an isomorphism $\left[ {\text {Tor}}^S_{j-1}(\Bbbk , I_\Delta )\right] _\textbf{b}\cong {\tilde{H}}^{|\textbf{b}|-j-1}(\Delta _\textbf{b}; \Bbbk )$. In conclusion,

4.5 $$\begin{aligned}{}[W_i(\Delta )]_\textbf{b}\cong {\tilde{H}}^{i-1}(\Delta _\textbf{b}; \Bbbk )^\vee , \end{aligned}$$

as soon as $|\textbf{b}|-i\geqslant 1$. $\square$

#### Remark 4.2

The isomorphism in the statement of Theorem 4.1 does not necessarily hold if we drop the hypothesis that $\textbf{b}$ is square-free. Indeed, $\left[ {\text {Tor}}^S_{|\textbf{b}|-i} (\Bbbk , \Bbbk [\Delta ])\right] _\textbf{b}$ is equal to 0 if $\textbf{b}$ is not square-free, [8, Theorem 8.1]. On the other hand, since the square-free multi-indices are finitely many, the vanishing of $\left[ W_i(\Delta )\right] _\textbf{b}$ for all $\textbf{b}$ that is not square-free implies $W_i(\Delta )$ is of finite length.

An alternate, less explicit proof of the above theorem can be obtained by applying the Bernstein–Gelfand–Gelfand correspondence to express $[W_i(\Delta )]_\textbf{b}$ as the (duals) of some ${\text {Tor}}$ spaces over the exterior algebra, and then apply a theorem of Aramova, Avramov, and Herzog [4], see [8, Corollary 7.5.2]. More precisely, we have the following result.

#### Proposition 4.3

For any $i\geqslant 1$ and any square-free multi-index $\textbf{b}$, there is a natural isomorphism of vector spaces

4.6 $$\begin{aligned} \left[ W_i(\Delta )\right] _\textbf{b} \cong \left[ {\text {Tor}}^E_{|\textbf{b}|-i} \bigl (\Bbbk , \Bbbk \langle \Delta \rangle \bigr )\right] _\textbf{b}^\vee . \end{aligned}$$

The proof of the proposition follows from an adaptation to the multi-graded context [5] of the classical BGG correspondence, as described in [7].

### Multigraded Hilbert series

Our next goal is to determine the Hilbert series of the Koszul modules $W_i(\Delta )$ associated to a simplicial complex $\Delta$. For a multidegree $\textbf{a}=(a_1, \ldots , a_n)\in \mathbb {N}^n$, we will write $\textbf{t}^\textbf{a} :=t_1^{a_1 }\cdots t_n^{a_n}$.

#### Theorem 4.4

For every simplicial complex $\Delta$ and every $i>0$, the $\mathbb {N}^n$-graded Hilbert series of the Koszul module $W_i(\Delta )$ is given by

$$\begin{aligned} \sum _{\textbf{a}\in \mathbb {N}^n} \dim _{\Bbbk } [W_i(\Delta )]_\textbf{a} \, \textbf{t}^\textbf{a}= \sum _{\begin{array}{c} \textbf{b}\,\in \, \mathbb {N}^n\\ \textbf{b} {\text {square-free}} \end{array}} \dim _{\Bbbk }({\widetilde{H}}_{i-1}(\Delta _\textbf{b}; \Bbbk )) \frac{\textbf{t}^\textbf{b}}{\prod _{j \in {\text {Supp}}(\textbf{b})}(1-t_j)}. \end{aligned}$$

#### Proof

We begin by observing that, by the definition of a square-free module, we have

4.7 $$\begin{aligned} \sum _{\textbf{a}\in \mathbb {N}^n} \dim [W_i(\Delta )]_\textbf{a} \, \textbf{t}^\textbf{a} = \sum _{\begin{array}{c} \textbf{b}\in \mathbb {N}^n\\ \textbf{b} \text { square-free} \end{array}} \dim [W_i(\Delta )]_\textbf{b} \, \frac{\textbf{t}^\textbf{b}}{\prod _{j \in {\text {Supp}}(\textbf{b})}(1-t_j)}. \end{aligned}$$

Thus, it suffices to determine $\dim [W_i(\Delta )]_\textbf{b}$ when $\textbf{b}$ is a square-free multidegree. Fix a square-free multidegree $\textbf{b}$, and let $j = |\textbf{b}| - i$. From Theorem 4.1, we know that

4.8 $$\begin{aligned}{}[W_i(\Delta )]_\textbf{b}\cong {\tilde{H}}_{i-1}(\Delta _\textbf{b}; \Bbbk ). \end{aligned}$$

Therefore,

4.9 $$\begin{aligned} \sum _{\textbf{a}\in \mathbb {N}^n} \dim [W_i(\Delta )]_\textbf{a} \, \textbf{t}^\textbf{a} = \sum _{\begin{array}{c} \textbf{b}\in \mathbb {N}^n\\ \textbf{b} \text { square-free} \end{array}} {\tilde{h}}_{i-1}\bigl (\Delta _\textbf{b}, \Bbbk \bigr ) \frac{\textbf{t}^\textbf{b}}{\prod _{j \in {\text {Supp}}(\textbf{b})}(1-t_j)}, \end{aligned}$$

and this completes the proof. $\square$

Specializing to the single $\mathbb {N}$-grading, the above theorem yields the following formula for the Hilbert series of the Koszul module $W_i(\Delta )$:

4.10 $$\begin{aligned} \sum _{a\in \mathbb {N}} \dim [W_i(\Delta )]_a \, t^a= \sum _{\begin{array}{c} \textbf{b}\,\in \, \mathbb {N}^n\\ \textbf{b}\, {\text {square-free}} \end{array}} \dim \bigl ({\widetilde{H}}_{i-1}(\Delta _\textbf{b}; \Bbbk )\bigr ) \left( \frac{t}{1-t}\right) ^{|\textbf{b}|}. \end{aligned}$$

In the particular case when $\Delta$ has dimension at most 1, that is, when $\Delta$ is equal to a (simplicial) graph $\Gamma$, we recover the Hilbert series of the module $W_\Gamma :=W_1(\Gamma )(2)$, as computed in [9, Theorem 4.1].

#### Corollary 4.5

[9] For a graph $\Gamma$ on vertex set ${\textsf{V}}$, we have

$$\begin{aligned} {\text {Hilb}}(W_{\Gamma },t )= \frac{1}{t^2}\cdot Q_{\Gamma }\Big ( \frac{t}{1-t} \Big ), \end{aligned}$$

where $$Q_{\Gamma }(t)=\sum _{j\geqslant 2} c_j(\Gamma ) t^j$$ and $$c_j(\Gamma )=\sum _{{\textsf{V}}'\subseteq {\textsf{V}}:|{\textsf{V}}'|=j } {\tilde{h}}_0(\Gamma _{{\textsf{V}}'})$$.

The significance of the above formula is that it gives the Chen ranks of the right-angled Artin group $G_{\Gamma }$ associated to the graph $\Gamma$.

## Resonance varieties of a simplicial complex

Given an (abstract) simplicial complex $\Delta$ on vertex set ${\textsf{V}}$, we define its resonance varieties as those of the corresponding exterior Stanley–Reisner ring. That is, we put $\mathcal {R}^i(\Delta ):=\mathcal {R}^i(\Bbbk \langle \Delta \rangle )$ for the jump resonance and $\mathcal {R}_i(\Delta ):=\mathcal {R}_i(\Bbbk \langle \Delta \rangle )$ for the support resonance varieties, respectively.

Using Proposition 3.8, a precise description of the varieties $\mathcal {R}^i(\Delta )$ was given in [10, Theorem 3.8], as follows.

### Proposition 5.1

For each $i\geqslant 1$, the decomposition in irreducible components of the jump resonance variety is given by

5.1 $$\begin{aligned} \mathcal {R}^i(\Delta )= \bigcup _{\begin{array}{c} {\textsf{V}}' \subseteq {\textsf{V}}\,\text {maximal such that}\\ \exists \sigma \in \Delta _{{\textsf{V}}\setminus {\textsf{V}}'}, \ {\widetilde{H}}_{i-1-|\sigma |} ({\text {lk}}_{\Delta _{{\textsf{V}}'}}(\sigma );\Bbbk ) \ne 0 \end{array}} \Bbbk ^{{\textsf{V}}'}. \end{aligned}$$

Here $\Bbbk ^{{\textsf{V}}'}$ denotes the coordinate subspace of $\Bbbk ^{{\textsf{V}}}=\Bbbk ^n$ (where $n=|{\textsf{V}}|$) spanned by the vectors $\{\textbf{e}_i \mid i\in {\textsf{V}}'\}$. On the other hand, for the support resonance defined in (2.10), the situation is different in degrees $i>1$.

### Theorem 5.2

For each $i\geqslant 1$, the scheme structure on the support resonance locus $\mathcal {R}_i(\Delta )$ is reduced. Moreover, the decomposition in irreducible components is given by

5.2 $$\begin{aligned} \mathcal {R}_i(\Delta )=\bigcup _{\begin{array}{c} \mathsf {V'}\subseteq {\textsf{V}}\, \text {maximal with}\\ {\widetilde{H}}^{i-1}(\Delta _{\mathsf {V'}};\Bbbk )\ne 0 \end{array}} \Bbbk ^{\mathsf {V'}}. \end{aligned}$$

### Proof

The first claim follows from Proposition 3.9 and Theorem 3.5. The precise structure of the decomposition in irreducible components is governed by the multi-graded structure detailed in Theorem 4.4 and Proposition 3.6. Observe that (5.2) corresponds to the primary decomposition of the ideal ${\text {Ann}}(W_i(\Delta ))$. $\square$

Notice the difference at the set level between (5.1) and (5.2); in particular, observe that the support resonance loci are easier to describe. Furthermore, whereas Theorem 5.2 guarantees that the support resonance schemes $\mathcal {R}_i(\Delta )$ are always reduced, the corresponding jump resonance loci $\mathcal {R}^i(\Delta )$ are not necessarily reduced (with the Fitting scheme structure), even in weight one, as the following example illustrates.

### Example 5.3

Let $\Gamma$ be a path on 4 vertices. Then ${\text {Fitt}}_0(W_1(\Gamma ))=(x_2)\cap (x_3) \cap (x_1,x_2^2,x_3^2,x_4)$ is not reduced, although ${\text {Ann}}(W_1(\Gamma ))=(x_2)\cap (x_3)$ is reduced. Therefore, the Fitting scheme structure on $\mathcal {R}^1(\Gamma )$ has an embedded component at 0.

A simplicial complex $\Delta$ of dimension *d* is said to be a *Cohen–Macaulay complex* over $\Bbbk$ if ${\widetilde{H}}^{\bullet }({\text {lk}}(\sigma );\Bbbk )$ is concentrated in degree $d -|\sigma |$, for all $\sigma \in \Delta$. As shown in [6], the jump resonance varieties of such a simplicial complex *propagate*, that is,

5.3 $$\begin{aligned} \mathcal {R}^1(\Delta ) \subseteq \mathcal {R}^2(\Delta ) \subseteq \cdots \subseteq \mathcal {R}^{d+1}(\Delta ). \end{aligned}$$

For arbitrary simplicial complexes, though, the resonance varieties do not always propagate. This phenomenon, first identified in [10], happens even for graphs.

### Example 5.4

[10] Let $\Delta$ be the disjoint union of two edges. Then $\mathcal {R}^1(\Delta )=\Bbbk ^4$, whereas $\mathcal {R}^2(\Delta )=\Bbbk ^2 \cup \Bbbk ^2$, the union of two transversal coordinate planes. Thus, $\mathcal {R}^1(\Delta ) \not \subseteq \mathcal {R}^2(\Delta )$.

When $\Delta$ is Cohen–Macaulay, propagation and formula (2.11) give $\mathcal {R}^i(\Delta )=\bigcup _{j\leqslant i} \mathcal {R}_j(\Delta )$. But it is not known whether the support resonance varieties $\mathcal {R}_i(\Delta )$ propagate when $\Delta$ is Cohen–Macaulay, or, equivalently, whether $\mathcal {R}^i(\Delta )=\mathcal {R}_i(\Delta )$ in this case. In general, though, we can use the previous example to settle the latter question in the negative.

### Example 5.5

Let $\Delta$ be the disjoint union of two edges. Then $\mathcal {R}_1(\Delta )=\mathcal {R}^1(\Delta )=\Bbbk ^4$ but $\mathcal {R}_2(\Delta )=\emptyset$ whereas, as we saw before, $\mathcal {R}^2(\Delta )=\Bbbk ^2 \cup \Bbbk ^2$. Thus, $\mathcal {R}_2(\Delta )\ne \mathcal {R}^2(\Delta )$.

## Regularity and projective dimension for Koszul modules of simplicial complexes

### General bounds

We start this section with an upper bound on the Castelnuovo–Mumford regularity and projective dimension of the Koszul modules.

#### Proposition 6.1

For every simplicial complex $\Delta$ on *n* vertices and every $i>0$, the Koszul module $W_i(\Delta )$ has regularity at most *n* and projective dimension at most $n-i-1$.

#### Proof

By definition, the Koszul module $W_i(\Delta )$ is a sub-quotient of the module $Z_i \subseteq {\bigwedge }^i V \otimes _{\Bbbk } S$ of *i*-th cycles in the Koszul complex of $x_1, \ldots , x_n$. Since $Z_i$ is generated in degree $i+1$, it follows that the degree of any of the generators of $W_i(\Delta )$ is at least $i+1$. Let $\textbf{F}_\bullet$ denote the minimal free resolution of $W_i(\Delta )$. By Proposition 3.9 and Corollary 3.3, $\textbf{F}_\bullet$ is a complex of $\mathbb {N}^n$-graded *S*-modules generated in square-free multidegrees, hence, the total degree of the generators of each $\textbf{F}_h$ is at most *n*. The statement on the regularity follows immediately. Since the least degree of the generators of $\textbf{F}_{h+1}$ is strictly larger than the least degree of the generators of $\textbf{F}_{h}$, it follows that $\textbf{F}_{h} = 0$ for $h > n-i-1$. $\square$

### Regularity of Koszul modules for simplicial complexes of special type

We fix integers $n\geqslant 4$ and $1\leqslant d\leqslant n-3$ and assume $\Delta$ is a simplicial complex of dimension *d* on *n* vertices whose $(d-1)$-skeleton coincides with that of the full simplex, that is,

6.1 $$\begin{aligned} \Delta ^{(d-1)}=\big (2^{[n]}\big )^{(d-1)}. \end{aligned}$$

For instance, if $d=1$, then $\Delta$ is simply a (simplicial) graph on *n* vertices. If $d=2$, then $\Delta$ is obtained from the complete graph on *n* vertices by filling in some triangles. For this type of simplicial complexes that generalize graphs, the nature of the Koszul modules can be made more precise, as follows.

#### Proposition 6.2

For a simplicial complex $\Delta$ as above, the following hold.

1. $$W_i(\Delta )=0$$ for $$i\notin \{d,d+1\}$$.
2. $$W_d(\Delta )={\text {coker}}\big (\partial ^E_{d+2}+j_{d+1}\otimes _{\Bbbk } S\big )$$.
3. $W_{d+1}(\Delta )=\ker \big (\partial ^A_{d+1}\big )$, and hence it is either zero or torsion-free.

#### Proof

Recall that $W_i(\Delta )=H_i (A_{\bullet } \otimes _{\Bbbk } S,\partial )$, where $A=\Bbbk \langle \Delta \rangle$. By condition (6.1), the graded algebra $A=E/J_{\Delta }$ satisfies the hypothesis from the beginning of Sect. 2.3. Hence, Proposition 2.2 applies, showing that $W_i(\Delta )=0$ for $i < d$. Moreover, since $\dim (\Delta )=d$, we have that $A_i=0$ for $i> d+1$, and so $W_i(\Delta )$ also vanishes in that range, thereby proving Part (1).

Part (2) follows at once from part (2) of Proposition 2.2, while part (3) follows from the fact that $A_{d+2}=0$. $\square$

Using the explicit presentation of $W_d(\Delta )$ from part (2), we can improve the bound on regularity from Proposition 6.1.

#### Proposition 6.3

With notation as above, we have ${\text {reg}}W_d(\Delta ) \le n-2$.

#### Proof

We have a presentation 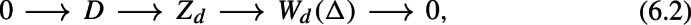 where $Z_d\subseteq {\bigwedge }^dV \otimes _{\Bbbk } S$ is the module of Koszul *d*-cycles, and *D* is the image of $K \otimes _{\Bbbk } S$ under the Koszul differential. Both $Z_d$ and *D* are generated in degree $d+1$. The module $Z_d$ has a linear free resolution, consisting of the truncated Koszul complex, so ${\text {reg}}Z_d =d+1$. Since the module *D* is square-free, its syzygy modules are also square-free, and hence, they are generated in degrees at most *n*. This implies that ${\text {reg}}D \le n-1$, since *D* does not have generators of degree *n*. Applying the long exact sequence of ${\text {Tor}}(-,\Bbbk )$ to (6.2), we obtain

6.3 $$\begin{aligned} {\text {reg}}W_d(\Delta ) \le \max ({\text {reg}}Z_d, {\text {reg}}D-1)= \max (d+1,n-2) = n-2. \end{aligned}$$

and this completes the proof. $\square$

If $d\leqslant 1$, that is, if $\Delta =\Gamma$ is a graph on *n* vertices, taking into account the degree shift, we obtain the bound

6.4 $$\begin{aligned} {\text {reg}}W_1(\Gamma ) \leqslant n-4. \end{aligned}$$

#### Example 6.4

If $\Gamma =\mathcal {C}_n$ is the cycle on $n\geqslant 4$ vertices, then the regularity of $W_1(\Gamma )$ attains the above bound:

$$\begin{aligned} {\text {reg}}W_1(\Gamma )=n-4 \quad \text {and} \quad {\text {pdim}}W_1(\Gamma )= n-2. \end{aligned}$$

This follows from (6.2), since in this case the module *D* has only one syzygy, of degree *n*.

#### Remark 6.5

For the Koszul module $W_d(\Delta )$, the simplified presentation (2.17) has the following nice interpretation. Let ${\widetilde{\Delta }}$ be the simplicial complex which is maximal (with respect to inclusion) among all simplicial complexes that share the same *d*-skeleton with $\Delta$; for instance, if $d=1$, then ${\widetilde{\Delta }}$ is the flag complex of the graph $\Gamma =\Delta$. Denote by $V_i$ the $\Bbbk$-vector space with the basis set of *i*-dimensional missing faces of ${\widetilde{\Delta }}$. We then have an exact sequence, 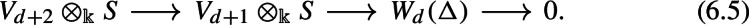


Using Proposition 6.2, together with formula (5.1), we obtain the following immediate corollary.

#### Corollary 6.6

With $\Delta$ as above, we have:

1. $$\mathcal {R}_i(\Delta )=\mathcal {R}^i(\Delta )$$ for all $$i\ne d+1$$.
2. $\mathcal {R}_{d+1}(\Delta )$ is equal to either $\emptyset$ or $\Bbbk ^n$.
3. $$\mathcal {R}^d(\Delta )= \bigcup _{\begin{array}{c} \mathsf {V'}\subseteq {\textsf{V}}\, \textrm{maximal}\\ {{\widetilde{H}}_{d-1}(\Delta _{\mathsf {V'}};\Bbbk )\ne 0} \end{array}} \Bbbk ^\mathsf {V'}$$.

#### Example 6.7

Let $\Delta$ be the boundary of the tetrahedron, with the face $\sigma =\{1,2,3\}$ missing. Then $\Delta ^{(1)}=\big (2^{[4]}\big )^{(1)}$, and so $\Delta$ is a simplicial complex covered by the above corollary, with $d=2$. In this case, we have that $\mathcal {R}_d(\Delta )=\{x_4=0\}$, since $H_1(\Delta _{\sigma };\Bbbk )=\Bbbk$, and $\mathcal {R}^d(\Delta )=\{x_4=0\}$, since ${\widetilde{H}}_{2-1-1}({\text {lk}}_{\Delta _{\sigma }}(\{4\});\Bbbk )={\widetilde{H}}_{0}(\emptyset ;\Bbbk )=\Bbbk$.

As already mentioned before, the loci $\mathcal {R}_{d+1}(\Delta )$ and $\mathcal {R}^{d+1}(\Delta )$ can be different, in general. For example, if we take the graph $\Gamma$ on four vertices with edges (1, 2) and (3, 4) as in Example 5.4, then $\mathcal {R}_2(\Gamma )=\emptyset$ while $\mathcal {R}^2(\Gamma )=V(x_1,x_2)\cup V(x_3,x_4)$.

## Footnotes

1 We denote by $\mathcal {R}^i(A)$ what in [11] is denoted by $\mathcal {R}^i_1(A)=\mathcal {V}^i_1(A^{\bullet } \otimes _{\Bbbk } S )$ and in [13] by $\mathcal {R}^i(A)$, whereas we use the notation $\mathcal {R}_i(A)$ for what in [11] is denoted by $\mathcal {W}^i_1(A)={\text {Supp}}H_i(A_{\bullet } \otimes _{\Bbbk } S)$ and in [13] by ${\widetilde{\mathcal {R}}}_i(A)$.
